# 克唑替尼治疗晚期或复发性ALK阳性非小细胞肺癌的疗效和安全性

**DOI:** 10.3779/j.issn.1009-3419.2019.08.02

**Published:** 2019-08-20

**Authors:** 晓燕 李, 华艳 许, 芳 高, 勋 康, 娟 张, 静 赵, 艺 林, 晓晴 刘

**Affiliations:** 1 100070 北京，首都医科大学附属北京天坛医院肿瘤综合治疗病区 Department of Oncologic Comprehensive Therapy, Beijing Tiantan Hospital, Capital Medical University, Beijing 100070, China; 2 100071 北京，解放军总医院第五医学中心肺部肿瘤科 Department of Lung Cancer, The Fifth Medical Center, General Hospital of People's Liberation Army, Beijing 100071, China

**Keywords:** 肺肿瘤, EML4-ALK, 克唑替尼, Lung neoplasms, EML4-ALK, Crizotinib

## Abstract

**背景与目的:**

间变性淋巴瘤激酶(anaplastic lymphoma kinase, *ALK*)基因在晚期非小细胞肺癌(non-small cell lung cancer, NSCLC)中的阳性比例约为5%-7%，ALK酪氨酸激酶抑制剂(tyrosine kinase inhibitor, TKI)为ALK阳性NSCLC患者的标准治疗。本研究拟评价克唑替尼在ALK阳性的晚期或复发性NSCLC患者中的疗效和安全性。

**方法:**

用三种方法筛选携带*ALK*基因融合/倒位的晚期或复发NSCLC患者，免疫荧光原位杂交(flourescence *in situ* hybridization, FISH)显示阳性者给予克唑替尼口服，250 mg，2次/d。评价客观有效率(objective response rate, ORR)、无进展生存期(progression-free survival, PFS)及安全性。

**结果:**

共筛选226例患者，其中39例出现*ALK*融合或倒位，37例入组，35例患者可评价客观疗效，ORR为70.3%，疾病控制率(disease control rate, DCR)为94.6%，中位PFS为11.8个月，主要不良反应为转氨酶升高(1级，91.7%)、转氨酶升高(2级，23.4%)、恶心(1级，75.6%)、贫血(1级-2级，62.3%)、视物障碍(1级，21.8%)、体重减轻(1级，31.4%)、肺炎(2级，3.5%)。

**结论:**

克唑替尼单药可用于治疗*ALK*融合/倒位的晚期NSCLC患者，有效率高，耐受性较好。

## Abstract (en)

**Background and objective:**

Anaplastic lymphoma kinase (ALK) positive in non-small cell lung cancer (NSCLC) was about 5%-7% and ALK tyrosine kinase inhibitor (TKI) was the standard treatment in NSCLC. The aim of this study is to evaluate the efficacy and safety of crizotinib in patients with advanced *ALK* gene-positive or recurrent NSCLC.

**Methods:**

Three methods were used to screen patients with advanced or recurrent NSCLC harboring *ALK* gene fusion/translocation. The patients with ALK positive tested by flourescence *in situ* hybridization (FISH) was given orally crizotinib, 250 mg, *bid*. The objective response rate (ORR), progression-free survival (PFS) and safety were evaluated.

**Results:**

A total of 226 patients were screened, 39 of whom had *ALK* fusion or translocation, and 37 were enrolled in the study. 35 patients were evaluated for objective response, ORR was 70.3%, and disease control rate (DCR) was 94.6%, and median PFS was 11.8 mon. The main adverse reactions were elevated transaminase (Grade 1, 91.7%), elevated transaminases (Grade 2, 23.4%), nausea (Grade 1, 75.6%), anemia (Grade 1-2, 62.3%), visual impairment (Grade 1, 21.8%), weight loss (Grade 1, 31.4%), pneumonia (Grade 2, 3.5%).

**Conclusion:**

Crizotinib can be used for the treatment of advanced NSCLC with *ALK* fusion/translocation. It is highly effective and well tolerated.

非小细胞肺癌(non-small cell lung cancer, NSCLC)是国内乃至全球死亡率最高的恶性肿瘤之一^[1]^，尽管5年生存率仍低于20%，但是近10年来，NSCLC的治疗有了突破性的进展，酪氨酸激酶抑制剂(tyrosine kinase inhibitors, TKIs)的出现并用于临床是其中之一。多项临床研究^[2-4]^已经证实，表皮生长因子受体(epithelial growth factor receptor, *EGFR*)敏感突变型NSCLC患者接受TKIs(如吉非替尼、厄洛替尼等)治疗，其中位无进展生存期(progression-free survival, PFS)可达10个月-12个月，中位总生存期(overall survival, OS)超过36个月，可谓是“里程碑式的进步”。继EGFR-TKI之后，棘皮动物微管相关蛋白样4-间变性淋巴瘤激酶(echinoderm microtubule associated protein like 4 gene-anaplastic lymphoma kinase, *EML4-ALK*)融合基因的发现及ALK抑制剂(如克唑替尼)的研究是NSCLC治疗的又一重要进展。

EML4-ALK最初在淋巴瘤中发现，其2号染色体的11号外显子发生断裂，并与*ALK*发生重组、融合^[5]^。陆续有报道在肺癌患者的肿瘤组织中发现*ALK*融合^[6, 7]^。2009年的美国临床肿瘤协会(American Society of Clinical Oncology, ASCO)年会上，Shaw^[5]^报告了Ⅰ期临床研究的结果：患有*ALK*融合或基因倒位的晚期NSCLC患者，给予ALK抑制剂——克唑替尼，其有效率达73%，中位PFS达9个月。基于这样良好的疗效和安全性，美国食品药品监督管理局(Food and Drug Administration, FDA)批准其直接进入Ⅲ期临床研究，其中A8081005研究在中国招募患者。解放军总医院第五医学中心南院区肺部肿瘤科为研究中心之一参加了005研究。本文将总结分析*ALK*融合患者经克唑替尼治疗后的客观有效率(objective response rate, ORR)、PFS及安全性。

## 1 资料与方法

### 1.1 一般资料

得到我中心伦理委员会批准以后，本研究自2011年1月开始入组，共筛选226例患者，含有*ALK*融合或倒位的39例，阳性率为17.3%，共37例接受克唑替尼单药治疗(250 mg, *bid*)，其中35例可评价客观疗效(2例患者在首次评价客观疗效前死亡)。截止2018年6月1日(距首例患者入组77个月)，37例患者中，1例患者仍在接受治疗，36例停止治疗，其中33例死亡。

### 1.2 入选/排除标准

(1) 入选标准：≥18岁的组织学或病理学确诊的局部晚期或转移性NSCLC; ALK分离免疫荧光原位杂交技术(fluorescence *in situ* hybridization, FISH)检测法显示*ALK*基因位点的异位或倒位阳性; 美国东部肿瘤协作组(Eastern Cooperative Oncology Group, ECOG)体力状况(performance status, PS)评分≤3分; 无症状的脑转移或存在脑转移，但经过治疗后稳定至少14 d以上; 既往的化疗、放疗在研究药物治疗开始前至少2周完成; 任何急性毒性反应必须已经恢复至≤1级(脱发除外); 足够的器官功能; 患者知情并理解研究方案，有能力遵循研究方案的访视计划; 患者及配偶同意采取有效的避孕措施。(2)排除标准：未接受过系统的抗肿瘤治疗(化疗或靶向治疗); 正在接受其他的临床试验治疗; 既往接受过针对ALK的治疗; 脊髓压迫、癌性脑膜炎或软脑膜疾病的患者; 不能控制良好的心血管疾病(心力衰竭、房颤、不稳定型心绞痛等)、妊娠期或哺乳期、合并使用已知是CYP3A4的强抑制剂; 近3年内，患者存在活动期恶性肿瘤; 已知患有间质性纤维化或间质性肺病的患者; 其他不适合参加本研究的情况。

### 1.3 *ALK*融合基因检测方法

采用FISH，由中心实验室进行检测。

### 1.4 给药、剂量调整及停药标准

给药：本研究供给的克唑替尼为片剂，共2个规格：100 mg、50 mg。口服，每日2次。每次服药时间记录于服药表。漏服、呕吐不再补服。不得研磨、碾碎或溶解后服用。

剂量调整：根据不良反应的严重程度，克唑替尼可进行两次剂量调整。

停药标准：若经过两次剂量调整后仍不能耐受，则予以停药。出现间质性肺炎需永久停药。客观评价疾病进展(progressive disease, PD)后，研究者判断患者仍可从克唑替尼中获益，则可继续服药，研究者判断患者不再从治疗中获益，则停药。患者拒绝继续服药时，随时停药。

## 2 结果

### 2.1 入组筛选及ALK阳性患者一般临床资料

ALK阳性的39例患者中，全部为亚裔，男性12例，女性27例; ≤50岁21例，> 50岁18例; 全部为腺癌患者; 32例患者从不吸烟，7例患者既往曾经吸烟; 18例患者EGFR野生型，1例外部机构检测为*EGFR* 21外显子L858R突变，我院复检结果为阴性，20例患者*EGFR*基因型不明确。

我们总结了37例可以评价客观疗效患者的临床病理学资料，从中可以看出：ALK阳性多见于女性、从不吸烟的腺癌患者; 入组时多为Ⅳ期; 超过1/3的患者之前接受过3个以上系统性治疗方案(包括化疗和EGFR-TKI靶向治疗); 超过2/3的患者PS评分较差。见表 1-表 2。

**1:** *ALK*融合基因患者的人口统计学数据 Demographic data of patients haboring *ALK* fusion gene

Index	Screening (*n*=226)	ALK^+^ (*n*=39) [*n* (%)]	*P*
Gender			0.054
Male	104	12 (11.5)	
Female	122	27 (22.1)	
Age (yr)			0.047
≤50	87	21 (24.1)	
> 50	139	18 (12.9)	
Pathological type			0.299
Adenocarcinoma	215	39 (18.1)	
Squamous	3	0 (0.0)	
Others	8	0 (0.0)	
Smoking status			0.006
Never smoker	139	32 (23.0)	
Ex or current smoker	87	7 (8.0)	
*EGFR* mutation			0.227
*EGFR* Mu+	13	0 (0.0)	
*EGFR* Mu-	94	18 (19.1)	
Unknown	119	21 (17.6)	
EGFR: epidermal growth factor receptor.			

**2:** 携带*ALK*融合基因患者的临床和病理特征(*n*=37) Clinical and pathological characteristics of the patients harboring *ALK* fusion gene (*n*=37)

Index	*n*	Ratio (%)	*P*
Gender			0.054
Male	12	32.4	
Fenale	25	67.6	
Age (yr)			0.385
20-30	1	2.7	
31-40	10	27.0	
41-50	8	21.6	
51-60	9	24.3	
≥61	9	24.3	
Smoking status			0.027
Never smoker	29	78.4	
Ex-smoker	6	16.2	
Current smoker	2	5.4	
Stage			0.016
Ⅲ or relapse	5	13.5	
Ⅳ	32	86.5	
ECOG PS			0.024
0	0	0.0	
1	11	29.7	
2	26	70.3	
Pathological type			0.001
Adenocarcinoma	37	100.0	
Squamous	0	0.0	
Others	0	0.0	
Brain metastasis			0.009
No	24	64.9	
Yes	13	35.1	
Number of prior regimens			0.145
1	12	32.4	
2	10	27.0	
≥3	15	40.5	
ECOG PS: Eastern Cooperative Oncology Group performance status.			

### 2.2 客观疗效及PFS

按照研究方案规定，使用实体瘤缓解评价标准1.1版(Response Evaluation Criteria in Solid Tumor 1.1, RECIST1.1)进行客观疗效评价。ALK阳性并接受克唑替尼治疗的37例患者中，2例在首次客观疗效评价前死亡，35例患者至少接受了1次客观疗效评价。其中确认完全缓解(complete response, CR)0例，部分缓解(partial response, PR)26例，疾病稳定(stable disease, SD)7例，PD(6周)2例。ORR为70.3%，疾病控制率(disease control rate, DCR)为94.6%。相对而言，女性患者、不吸烟患者及既往接受更多治疗的患者，其ORR更高(分别为72.0% *vs* 66.7%、72.4% *vs* 62.5%)。截止数据分析时，共有1例患者仍未观察到PFS，余36例患者的PFS为3个月-60个月，中位PFS为11.8个月)，其中8例患者因严重不良事件永久停药，余28例患者中有6例在PD后因研究者判断仍有临床获益，继续服用克唑替尼。6例PD后继续服药的患者，继续用药时间为12周-42周，均评价为再次PD后停止服药。见表 3-表 5。

**3:** *ALK*融合基因患者的疗效 Clinical efficacy of patients haboring *ALK* fusion gene

Index	*n* (%)
Not done^1^	2 (5.4)
CR	0 (0.0)
SD	7 (18.9)
PR	26 (70.3)
6 wk	23 (88.5)
12 wk	3 (11.5)
PD (6 wk)	2 (5.4)
Dramatic progression	2 (5.4)
Gradual progression	0 (0.0)
Local progression^1^	0 (0.0)
1: 2 patients died before first assessment. CR: complete response; SD: stable disease; PR: progressive disease; PD: progressive disease.	

**4:** *ALK*融合基因患者的客观有效率 Objective response of patients haboring *ALK* fusion gene

Index	ORR (*n*/*N*)
Number of prior regimens	
1	66.7% (8/12)
2	50.0% (5/10)
≥3	86.7% (13/15)
Gender	
Male	66.7% (8/12)
Female	72.0% (18/25)
Smoking status	
Never smoker	72.4% (21/29)
Ex or current smoker	62.5% (5/8)

**5:** *ALK*融合基因患者的无进展生存期 Progression-free survival of patients haboring *ALK* fusion gene

Index	Median PFS (wk) (interval)
Gender	
Male	19 (12-52)
Female	24 (6-92)
Age (yr)	
≤50	32 (12-70)
> 50	28 (6-92)
Response	
PR	32 (12-92)
SD	32 (12-48)

### 2.3 总生存期(overall survival, OS)

接受随机治疗的37例患者中，已有20例死亡，可计算的OS为3个月-63个月，中位OS为35个月。分析发现，性别、吸烟与否等与OS无显著相关。

### 2.4 不良事件及安全性

在患者服用克唑替尼期间，共记录不良事件173例次，血液学毒性主要表现为中性粒细胞减少和贫血，非血液学毒性主要表现为转氨酶升高、视觉障碍、恶心。多为Ⅰ级-Ⅱ级(94.2%)，1例患者因出现3级中性粒细胞减少而暂时停药，恢复后减量至200 mg，*bid*; 1例患者因出现3级谷丙转氨酶(alanine aminotransferase, ALT)升高而暂时停药，降至ALT升高1级后减量至200 mg，*bid*。见表 6。

**6:** *ALK*融合基因患者的不良事件和安全性 The adverse events and safety of patients haboring *ALK* fusion gene

Toxicity	Ⅰ	Ⅱ	Ⅲ-Ⅳ
Hematologic			
Neutrophilic granuloaytopenia	16	7	2
Thrombocytopenia	5	3	0
Anemia	17	5	0
Non-hematologic			
ALT elevation	32	4	1
Visual disturbance	21	0	0
Pneumonitis	0	0	6
Nausea	14	5	0
Emesis	6	1	0
Diarrhea	2	0	0
Prolonged QTc	8	0	0
Bradycardia	8	0	0
Dysphagia	7	0	0
Hypotention	2	0	1

### 2.5 严重不良事件

截至2018年6月1日，本研究共报告严重不良事件(serious adverse events, SAE)10例，其中6例为重症肺炎，患者快速发展为呼吸衰竭而死亡，目前尚无充分证据证明重症肺炎与研究药物克唑替尼有关。另外4例SAE分别为脑出血、截瘫、呼吸衰竭、休克，最终结局均为死亡，均因病情进展出现肿瘤相关症状，与研究药物克唑替尼无关。见表 7。

**7:** ALK阳性患者的严重不良事件 Serious adverse events (SAE) of ALK positive patients

	Sceening number	Gender	Treatment duration	SAE	Relationship with crizotinib	Discontinue treatment or reduce dose
1	12171021	Male	16 wk	Pneumonitis	Unknow?	Discontinue
2	12171041	Female	4 wk	Pneumonitis	Unknow?	Discontinue
3	12171049	Male	18 wk	Pneumonitis	Unknow?	Discontinue
4	12171056	Female	16 wk	Pneumonitis	Unknow?	Discontinue
5	12171098	Female	26 wk	Pneumonitis	Unknow?	Discontinue
6	12171134	Female	26 wk	Pneumonitis	Unknow?	Discontinue
7	12171127	Male	48 wk	Cerebral hemorrhage	No relationship	Discontinue
8	12171153	Female	12.5 wk	Paraplegia	No relationship	Discontinue
9	12171210	Male	32.5 wk	Respiration failure	No relationship	Discontinue
10	12171059	Female	53 wk	Shock	No relationship	Discontinue

## 3 讨论

EML4编码蛋白N-末端部分融合至ALK的细胞内酪氨酸激酶结构域，重排为*EML4-ALK*，导致异常酪氨酸激酶表达。2007年Soda^[6]^首次在NSCLC患者术后标本中检测到*EML4-ALK*重排融合。此后，美国、日本、韩国及中国香港均有报道^[7-9]^，但在未经选择的NSCLC中，*EML4-ALK*阳性检出率较低，约1.5%-6.7%。为了提高该融合基因的检出率，研究入组的患者必须具备两种或更多的下列特征：女性、亚裔、无或仅少量吸烟史、腺癌。结果显示141例患者中19例(13%)为*EML4-ALK*阳性，如果不吸烟或仅少量吸烟者/和不伴有*EGFR*突变者，*EML4-ALK*阳性率分别高达22%和33%，这也是迄今报道*EML4-ALK*检出率最高人群。我中心筛检的参加005研究的患者，ALK阳性率为17.3%，高于吴一龙等^[10]^、陈海泉等^[11]^的报告(分别为6.4%和5.7%)。通过对相关因素的分层分析，可以发现：NSCLC患者中，女性、腺癌、不吸烟、EGFR野生型患者更容易出现*ALK*融合。

克唑替尼是ALK/c-MET小分子抑制剂，自2010年ASCO年会首次报告克唑替尼的Ⅰ期临床结果以来，因其较高的有效率及良好的耐受性而备受关注。Ⅱ期和Ⅲ期临床研究的结果进一步巩固了Ⅰ期试验的结论，因此2011年8月26日美国FDA批准克唑替尼(Xalkori)上市。2011年ASCO年会上报告了005研究的中期研究结果，更进一步证实了克唑替尼在复治和复发且含*ALK*融合的晚期NSCLC患者的有效性和安全性。*ALK*融合的患者可一线给予克唑替尼治疗作为Ⅰ类推荐已被写入NCCN指南。

克唑替尼相关的毒性主要表现为转氨酶升高、恶心、贫血、中性粒细胞减少及视物障碍，多为1级-2级，恶心及视物障碍多可自行恢复，轻度转氨酶升高及贫血可适当给予口服药物。本研究有2例患者出现3级-4级ALT升高，分析发现，这2例患者均有肝脏转移瘤，按照研究方案暂时停药并给予对症治疗，降至正常范围或仅1级升高后，克唑替尼予以减量(200 mg, *bid*)，后ALT未再出现≥3级升高，考虑肝转移和克唑替尼两个因素加重了肝脏损害，但均不属于Hy’s Law(药物引发的肝损伤)。

Tamiya^[12]^和Watanabe^[13]^分别报道了克唑替尼导致的间质性肺炎，我中心发生的10例SAE中，6例为重症肺炎，病例特点为：(1)不发热，发病突然，常见症状为咳嗽与呼吸困难; (2)影像学主要表现为：双肺多发渗出性斑片影; 多与肿瘤所在段、叶无关; (3)常规抗生素治疗效果欠佳，2例痰培养发现致病菌，4例痰液及分泌物无阳性发现; (4)激素治疗欠佳; (5)病情发展迅速，均发展为呼吸衰竭而死亡。因发生肺炎后无病理诊断，且影像学不十分支持间质性肺炎的表现，故尚不明确这6例肺炎的发生是否与克唑替尼有关。

综上，作为小分子TKI，克唑替尼在*ALK*融合性肺癌中表现了良好的疗效和安全性，在临床应用中，其安全性将受到更严格的监控。
